# Understanding parents’ views toward the newly enacted HPV vaccine school entry policy in Puerto Rico: a qualitative study

**DOI:** 10.1186/s12889-021-11952-w

**Published:** 2021-10-25

**Authors:** Vivian Colón-López, Diana T. Medina-Laabes, Roxana Soto Abreu, Olga L. Díaz Miranda, Ana P. Ortiz, María E. Fernández, Pamela C. Hull

**Affiliations:** 1 Comprehensive Cancer Center-University of Puerto Rico, Cancer Control and Population Sciences, San Juan, Puerto Rico; 2 Department of Health Services Administration, Graduate School of Public Health, University of Puerto Rico Medical Science Campus, San Juan, Puerto Rico; 3 Department of Epidemiology and Biostatistics, Graduate School of Public Health, University of Puerto Rico Medical Science Campus, San Juan, Puerto Rico; 4 Department of Health Promotion and Behavioral Sciences, University of Texas Health Science Center, Houston, Texas United States; 5 The University of Kentucky, Department of Behavioral Science, College of Medicine, Markey Cancer Center, Lexington, Kentucky United States

**Keywords:** HPV vaccine, School entry policy, Parental perspective, Hispanic

## Abstract

**Background:**

The Human papillomavirus vaccine (HPV) is an essential tool for the prevention of HPV-related cancers. In Puerto Rico, the Secretary of Health established a school entry requirement of at least one dose of HPV vaccination in girls and boys aged 11 and 12 years, taking effect in August 2018. Our study aimed to examine parents’ and guardians’ views of unvaccinated children about the process of implementation of the new HPV vaccination school entry policy in Puerto Rico and identify potential barriers and facilitators related to the implementation of this requirement.

**Methods:**

During April through November 2019, we conducted three focus groups (*n* = 12) and eight in-depth semi-structured interviews with parents of children aged 11 and 12 who had not yet initiated the HPV vaccine series. The interview topics addressed were: perception of vaccination, HPV vaccine and it is inclusion as new school entry requirement practice, procedure of the sources of information, influencers, and willingness to change. The interviews were recorded and transcribed by our staff members. We identified emergent themes through thematic analysis.

**Results:**

The participants’ perspective on the HPV vaccine school requirement was mixed. Lack of information of the HPV vaccines and lack of communication about the school-entry requirement were the themes most mentioned in the interviews. Moreover, previous negative experiences from friends or family members and adverse effects deterred some participants from vaccinating their kids. We discussed barriers in the process of soliciting an exemption.

**Conclusion:**

Most barriers mentioned by study participants are modifiable. Information about the HPV vaccine mandate’s implementation and educational materials regarding HPV vaccine safety need to be provided to address parents’ concerns related to the vaccine’s side effects. Schools (teachers, principal directors, and administrative staff), the government, and parent organizations need to be part of these efforts. This multilevel approach will help to improve disseminating information about HPV vaccination to clarify doubts and misinformation among parents.

**Supplementary Information:**

The online version contains supplementary material available at 10.1186/s12889-021-11952-w.

## Background

The HPV vaccine is a crucial tool for prevention against human papillomavirus (HPV) and its related cancers. In 2018, the World Health Organization (WHO) made a Call for Action to eliminate cervical cancer. This plan includes 90% of the girls being full vaccinated with HPV vaccine by age 15 years in 2030 [1]. A similar strategy has been promoted by *HealthyPeople 2030* in the United States (US), which their goal is to achieve that 80.0% of adolescents aged 13 through 15 years received recommended doses of the HPV vaccine [2]. However, recent data from the 2019 National Immunization Survey showed that adolescents aged 13–17 were 54.2% up-to-date with the immunization series for HPV vaccine in the US [3]. This result implies that the national HPV vaccine uptake remained far from the goal.

As an evidence-based strategy, school-entry requirements have proved to increase vaccination rates among children and youth [4]. The Advisory Committee on Immunization Practices (ACIP) recommends the HPV vaccine as a routine vaccine for girls (since 2006) and boys (since 2011) at ages 11–12 years old [5]. Five US states or territories (Virginia, District of Columbia, Rhode Island, Puerto Rico, and Hawaii) implemented the HPV vaccine as a requirement for school entrance on those entering to 6 or 7 grades or started 11–12 years old [6]. Even with these efforts at the policy level, parents’ negative beliefs and attitudes on childhood vaccination still remains a challenge [7].

Vercruysse and co-authors (2016) [8] found that most parents from Northeastern states agreed with the school-based HPV vaccination program, but they showed weak support for making the vaccine a school entry requirement. In this qualitative study, parental choice and the unlikely casual-contact transmission of HPV in the school setting were their primary reasons against the mandate. A study in Connecticut in 2016 reported that parents did not expressed opposition to a hypothetical HPV vaccination requirement and perceived that it would be similar to other routine vaccines for school entry while also adding the benefit of cancer prevention [9]. To our knowledge, only one qualitative study [10] evaluated the parents’ perception of HPV vaccination mandate in Virginia, a state with HPV vaccine requirement since 2009 [11]. Pitt et al. 2013 [10] found resistance to the HPV vaccine mandatory from some parents resulting in parental autonomy as the main barrier, followed by concern about the safety of this vaccine [10]. Notably, few qualitative studies have evaluated the parents’ perceptions regarding mandatory HPV vaccination for school entry in US states and territories.

In Puerto Rico (PR), cervical and oropharyngeal cancer (in men) are the most common HPV-related cancers [12]. Also, a recent study observed an increase in cervical cancer after evaluated the period 2001–2017 [13]. Under the PR’s Law #25 from September 25, 1983 (the Immunization Law), the PR government grants to the Puerto Rico Secretary of Health (SHPR) the authority to decide which vaccine will be required for school entry [14]. In August 2018, the SHPR established at least one dose of the HPV vaccine was required for school entrance for girls and boys aged 11 and 12 years [15] to prevent and reduce the incidence of HPV-related cancers in PR. Annually, the SHPR has amended this requirement for each school year (2018–2019: 11–12 years old; 2019–2020: 11–14 years old, and 2020–2021: 11–16 years old) [16]. The purpose of this study was to examine the parents’ and guardians’ perception of unvaccinated children about the process of implementation of the new HPV vaccination school entry policy in PR, and identify potential barriers and facilitators related to this requirement.

## Methods

### Study design and sample

The implementation of school-entry policy for HPV vaccination (HPV-PIVac study) is a prospective study to understand geographic variation in HPV vaccine policies and outcomes across US states and territories, and studying the implementation and impact of the new school-entry HPV vaccine policy in PR. As part of this study**,** we conducted a a qualitative study between April to November 2019, 8–15 months after the HPV school-entry policy took effect in PR. Eligible criteria were: (1) parents or guardians (i.e., participants) (2) of boys and girls aged 11 and 12 years old who have not initiated the HPV vaccine series at the time of the interview, and (3) lives in San Juan or surrounding municipalities (Carolina, Trujillo Alto, Guaynabo, Caguas or Aguas Buenas).

We recruited 20 participants for the focus groups of a total of 40 screened. Fifty percent of those screened did not participate in the study because of time availability, loss of contact, or they vaccinated their child before the interview. All the recruited participants were women between the ages of 23 to 68 years old and mainly residents of the municipality of San Juan (80%); 18 reported being mothers and two grandmothers. Twelve participants were interviewed in focus groups (two focus groups, each with 2 participants and one focus group with 8), and eight participants were in-depth interviews.

### Recruitment

We used a purposive sampling. Several strategies were used to recruit the study participants. First, we posted flyers with information from the study in clinical settings, including Federally Qualified Health Clinics, pediatric clinics, and hospitals. Also, we distributed flyers to parents in several activities in the community and schools (sports, health fair, organic food fair, and social activities). We briefly discussed face-to-face with them (if requested), information of the study (study objectives), and their expected role in this study. We asked for contact information among those who were interested. Moreover, we conducted door-to-door outreach in public housing complexes. Their community leaders and the social workers collaborated to identify potential participants. All these venues were located in San Juan and surrounding municipalities. Lastly, we posted recruitment materials on Facebook to promote the study and increase engagement. A staff member screened those potential participants interested in our study by phone to determine their eligibility by a staff member. We scheduled the interview according to the participants’ time availability and the dynamics that made them feel more comfortable to speak (focus group or in-depth interview). Data were not affected because we used the same guideline for both methods, focus group and in-depth interview.

### Data collection procedures

A trained staff discussed the informed consent one-to-one with the participants, by telephone or in-person. All participants signed the informed consent. The participants who preferred telephone interviews, informed consent was sent by email.

#### Questionnaire

Before the interview, a questionnaire, in Spanish, with closed-ended questions were administrated to each participant to gather parent’s demographic information (gender, age, marital status, level of education, income and religious preference); children’s information (gender, health insurance, type of school and vaccination record); awareness of the HPV vaccine and its inclusion as school-entry vaccine requirement. The questionnaire was developed and collected in Qualtrics (SAP, Utah/Seattle, US).

#### Focus group and in-depth interview

We used the same semi-structured interview guideline for the focus groups and in-depth interviews (see supplementary document), with open-ended questions that prompted participants to discuss the following topics: perception of vaccination in general, HPV vaccine, and the new school entry requirement policy; practice and procedure of the sources of information; influencers; and willingness to change. We gave fictitious names to those participants on the focus group and a random number for those who participated in in-depth interviews to protect their identities. Both focus groups and in-depth interviews were conducted in Spanish by the training staff. Focus groups lasted 40–60 min, and the in-depth interviews lasted around 20–40 min. Of three focus groups, two were held in a private conference room in the University of Puerto Rico Comprehensive Cancer Center (UPRCCC). The other focus group was conducted in a private room in a public housing complex. Two trained staff were in each focus group. One was responsible for guiding the interview, and another person to take notes. The in-depth interviews were face-to-face (in the UPRCCC) or over the phone, using the same dynamic as the focus group regarding the staff. Participants received $25.00 to compensate for their time. Incentives were provided in-person or sent by certified postal mail. All the data collected through the interviews (focus groups and in-depth) were audio-recorded and transcribed verbatim in the same language as the interview. A separate staff member reviewed the transcriptions for validation. For this publication, quotes were translated into English.

### Data analyses

Qualitative data management and analysis were performed using Atlas.ti 8.0 (Scientific Software Development GmbH, Berlin, Germany). We used thematic analysis to identify, analyze, and report themes [17]. First, one of the members (DML) performed the initial coding, and another member (VCL) reviewed the identification of the codes derived from the data and the quotes linked to the codes. Second, both members reviewed the codes several times until they met a consensus to define them and determine the emerging codes [18, 19]. We evaluated each interview separately for the coding process, and then we combined them to classify the codes by theme. Twenty-three codes were identified and organized into five themes (Attitudes and Beliefs, Knowledge, Communication, Barriers, and Recommendations). We used descriptive statistics to summarize the quantitative data from the questionnaire.

## Results

According to the questionnaire, most participants reported having less than some college education (70.0%) and an annual income of less than $15,000 (68.4%). The vast majority of the participants’ children were enrolled in public school (80.0%) and had Medicaid government health insurance (70.0%). The majority (80.0%) of the participants heard about the HPV vaccine, mainly through health providers. Despite this, over half of them (55.0%) were unaware that the HPV vaccine was a school-entry requirement. In addition, 13 of 20 participants reported that they vaccinated their children with all the required vaccines for school except HPV vaccine (Table 1).

**Table 1:** Characteristics of Focus Groups and In-Depth Interviews Participants: HPV-PIVac Study 2019 (*n* = 20)

Characteristics	Frequency n (range or %)
Age (mean)	41 (range: 23–68)
Education	
Some college or more	6 (30.0)
Less than some college	14 (70.0)
*Income*^*α*^	
≥ $15.000	6 (31.6)
< $15.000	13 (68.4)
*School*	
Public	16 (80.0)
Private	4 (20.0)
*Child’s health insurance*	
Medicaid	14 (70.0)
Private	5 (25.0)
Non-insurance	1 (5.0)
*How important is your religious beliefs?*	
Important or very important	16 (80.0)
Little important or unimportant	4 (20.0)
*What required vaccine the children had, except HPV vaccine?*	
*All of them* ^*α*^	13 (65.0)
*Some of them*	7 (35.0)
*Who told you about HPV vaccine?* ^*β*^	
Health Provider	9 (45.0)
School	7 (35.0)
Family member	4 (20.0)
TV commercial	8 (40.0)
Unknown about the HPV vaccine	4 (20.0)
*Did you know that HPV vaccine was included as vaccination requirement for starting school?*	
No	11 (55.0)
Yes	9 (45.0)
*If the HPV vaccine is required for the entry school, as parent, what will you do?*	
Vaccinate my son/daughter	9 (45.0)
Depending on the doctor recommendation	4 (20.0)
Sign a vaccine exemption	5 (25.0)
Need more information to take a decision	2 (10.0)

α: Tdap, meningococcal vaccine, Polio, MMR, VAR, Hib, Hepatitis B

β: the participants chose more than one option

### Participants’ attitudes and beliefs mixed regarding HPV vaccine school entry policy

In general, almost all the participants stated that vaccination is a positive preventive method against diseases, although few had doubts about their efficacy and safety. Participants’ perception of including the HPV vaccine as a school entry requirement was mixed. Half of the participants expressed being in favor. Some participants mentioned that this policy might ensure early protection against the HPV infection or future cancer development. Two participants (grandparents) agreed with the HPV-school entry requirement. These guardians witnessed in the past the mortality caused by now preventable diseases due to limited access to treatment.

Those who hesitated to the HPV vaccine requirement mentioned different reasons: administration at an early age, distrust in the efficacy and safety of the vaccine, and parental autonomy. Participants hesitated by the age (11–12 years old) of the mandate did not wholly reject the intention to vaccinate their children. Instead, they thought of vaccinating their children when they get older.

*“I think that if he is not sexually active … I think that [when] he turns 15 years old, he can be [vaccinated]. [W] hen he [can] understand, and we can talk about that topic. [A] 11 and 12 [year-old] boy [does] not [understand] what the vaccine is for or what it uses for.”*

The participants commonly expressed previous negative experiences with the HPV vaccine or other vaccine from friends or family members in the interviews. In some participants, these anecdotes caused them uneasiness about the safety and effectiveness of the HPV vaccine since it was a “new” vaccine for them. Other participants disclosed their children’s pre-existing medical conditions, indicating their concerns about how the vaccine’s components and its potential adverse effects could exacerbate their children’s health condition.

### HPV vaccine knowledge and unawareness about the HPV school entry policy

Most of the participants were aware of how many doses are required to complete the series and that HPV is a sexually transmitted disease. Few mentioned the association between this vaccine and cancer prevention. Despite their knowledge about the vaccine, some participants indicated that they had never heard about the new HPV vaccine school entry requirement. One participant expressed the constant promotion of this vaccine through the mass media (TV, Facebook, newspaper). Still, she never noticed it in the immunization record (commonly known in PR as the ‘*green sheet’*) required for the school entry.

*“A lot of promotion about the [HPV] vaccine, but I did not know that it was mandatory to vaccinate them for school, [I mean], that it existed in the green sheet of vaccines …*. “.

### Lack of communication: the main barrier for school-entry policy

The participants’ main barrier regarding the HPV vaccine requirement policy was the communication between the parents, the schools and health provider. Participants expressed that the information provided by the school, mainly during the school enrollment process, was general in content: a paper with a list of all the materials, vaccination requirements, and a request of a medical evaluation if it applies. They also argued that their children’s schools lacked educational opportunities or a space for explaining or clarifying doubts about the vaccine requirement. Only two participants remembered receiving a notification from the school about the HPV vaccine being including in immunization requirements. Some participants expressed that the school nurse called them to remind them about the deadline to vaccinate their child without further explanation.

Although most participants indicated that they received information about the HPV vaccine and its school requirement from their healthcare providers, most of them verbalized that the experience was insufficient. Some participants said that the only information they received from pediatricians was that “*it is mandatory*” or “*you need it for school*”.

*“[When] you go to a pediatrician’s office, the only thing that they tell you is that you have to give [to your child] the vaccine, and that’s it!”*

In contrast, participants who decided to request a medical exemption indicated that their providers talked to them about vaccination. In this conversation, the provider explained the pros and cons of the HPV vaccine and whether the child’s condition could be compromised after the vaccine administration. One participant felt that her privacy was respected when her health provider talked with her because it was a ‘closed-door’ conversation and he did not judge her.

Almost all the participants discussed the TV advertisements seen regarding the HPV vaccine. For some participants, these TV advertisements were the only source to get information about the HPV vaccine. Others expressed that the sense of guilt the advertisement promoted made them feel blame for their children’s future.

*“TV blames you and makes you a bad parent. The ad blames you and makes you a bad parent.”*

Interestingly, this advertisement made some of them think to eventually vaccinate their children because if they can prevent something wrong happened to their child, they will do it. Moreover, Facebook and the internet were the tools used by these participants to find information about vaccines. However, they stated that they were aware that the information found on social media and websites would not necessarily be accurate.

### Barriers regarding HPV vaccine and exemption

The lack of information about the HPV vaccine was another barrier identified. The main topics regarding lack of information were the pros and cons of the HPV vaccination to be required, and concerns about what happened if the vaccination series is interrupted. Other concerns expressed were alleged associations with autism, paralytic effects, an allergic reaction, and deathly consequence. Most of the participants said that seeing, listening, or reading this kind of (mis) information made them mistrust the HPV vaccine.

*“I talked with other parents and hearing news too, and all those things, like increasing [my doubts] and make me not want to allow [my child] to get the vaccine.”*

Access to the vaccine, availability to coordinate an appointment, and the cost of getting the immunization record signed by the health provider were also barriers mentioned. Two participants mentioned the high price of the vaccine as a general issue but not directly impacted. Three participants indicated that they went through the process of obtaining a medical exemption for the HPV vaccine. Complexity and the cost of the process affected their intent to get the exemption. They explained that it was a tedious and bureaucratic procedure to identify a healthcare provider or religious leader who would attest to the exemption and then find and coordinate an appointment with a lawyer to sign the affidavit. Exemptions from vaccination require to be renewed every year in PR. Therefore, parents had to incur the cost and go through these efforts every year.

*“ If it’s necessary, I’ll do it again [to avoid] putting things in my daughter that I do not consider necessary.”*

### Recommendations

The participants did not mention facilitators in their interviews. However, they recommended two primary information sources: schools and health care providers. Both should educate about the HPV vaccine and its requirement for school entry. The participants suggested that schools should be more proactive in providing educational sessions for parents and students whenever there are new changes to the requirements for school-entry. The participants said that health care providers, specifically those who administer vaccines, should be aware of the components of vaccines, their benefits, and potential adverse effects to explain this information to parents who have questions. Other recommendations from the participants were the sources’ message should not focus on using the fear factor, and to use social media and mass media to inform the community.

*“[In] the orientation, the pros and cons have to be indicated but not using the fear factor to impose or obligate a person to vaccinate [her/his] child*.”

## Discussion

In August 2018, PR was the 4th state/territory of the US to enact an HPV vaccine mandate for school entry [6]. We collected our data a couple of months after the Puerto Rico Department of Health (DOH) implemented this new policy. According to our results, in this group of parents and guardians of unvaccinated children, their views regarding the HPV vaccine school policy were mixed. Participants mentioned the vaccine’s benefits (i.e., prevention for cancer and HPV infection), and others said they disagreed because of the policy’s age and the mandatory aspect. The most emergent themes were a lack of communication about the school entry policy and information about the HPV vaccine. Despite the participants not mentioning any facilitator related to implementing the HPV vaccine school requirement during their interviews, they all indicated their recommendations.

Early age, parental autonomy, and concerns about vaccine safety were reasons that some hesitant participants mentioned in disbelief of the vaccine and the implementation. Hispanic parents recruited from other studies expressed this same concern of the age recommended for the HPV vaccine and often linked it with sexual activity [20–22]. ACIP recommended administration of HPV vaccine at 11–12 years old because it showed a robust immune response [5]. The scientific literature did not find evidence among the reviewed of several studies regarding an association between the HPV vaccine and promiscuity [23, 24]. Congruent to our qualitative analysis, 45% of our participants reported interest to vaccinate their child against HPV vaccine if it was a requirement. These results are consistent with a systematic analysis showed that parents’ agreement with the HPV school-entry mandate ranged from 27 to 63.5% [25]. Those against the potential future mandate stated the following arguments: limited information about the requirement, age, and lack of parental autonomy and those in favor were regarding prevention [25]. These arguments are similar to our participants’ opinions. However, the studies included in this analysis were conducted almost a decade ago. Also, one was in Guyana and the others were in states of the US (Georgia, Massachusetts, and Mississippi) who had not the HPV-school entry policy [10].

Vaccine hesitancy is defined as either refusal, delay in getting vaccinated or accepting vaccination with concern [26, 27]. In our study, the hesitation to the HPV vaccine among participants did not necessarily translate to hesitation about other adolescent immunizations or refusal of all required vaccines. Thirteen of the 20 participants reported that their children had all the other vaccines required for the school, except the HPV vaccine. More than half of our participants were unaware of the policy; despite the DOH providing information to the public a year prior (June 2017) [14]. The lack of awareness and difficulty accessing the HPV vaccine were the other reasons for some of our participants have not vaccinate their child at the time of the interview.

All our participants mentioned personal stories or negative experiences from someone regarding the adverse effects of the vaccines. MacDonald and co-authors [26] have suggested that factors associated with vaccine hesitancy are individual and group influence based on personal perception, personal experience, and the social media. Previous negative experiences with a vaccine could lead a parent to be hesitant and mistrust the effectiveness and safety of the vaccination in general [28]. For vaccine requirements, these negative vaccination experiences could directly affect the compliance of the mandate. Unfortunately, when these stories get disseminated via social media, people rely more on anecdotal, experience-based information than evidence-based information [7]. Besides, the spread of misinformation through these media could get more impact due to easy access. Understanding the scope and variability of the current sentiment toward the HPV vaccine and its policy could inform the development of an education campaign to combat the harmful misinformation using these same delivery channels [29, 30].

The lack of communication between the parents and guardians with the school and health providers was the main barrier to the HPV vaccine school entry policy. Similar to our studies [21, 30, 31]*,* parents have recommended workshops in a school setting given the accessibility. Having trained school personnel and integrate parents and students, the school could improve the engagement with the HPV vaccine policy [32]. Therefore, assess the school staff’s role (e.g., the school nurse, school principal, teachers), attitude, beliefs, and impact in vaccination school policy could strengthen the importance of integrating them in these preventive efforts. Regarding the communication with the health providers, several participants described it as insufficient. According to the participants’ discussion, the health provider mentioned that the HPV vaccine was mandatory for school entry without more explanation. The literature suggests that insufficient or inadequate communication between parents and health providers could negatively influence parents’ decisions and contribute to hesitant behavior [33, 34]. The influence of the healthcare providers’ recommendation has on the parents’ decisions on vaccination uptake (in general) is constantly reported [33–37]. This is also consistent with a previous household survey in PR conducted prior to the requirement (2008), in which 89% of parents of daughters in vaccine recommended age groups reported that they would vaccinate them if the doctor recommended it [37]. Strategies continually developed for the healthcare providers could provide effective communication and build trust with parents to recommend vaccination [34, 38].

In our studies, the lack of information was a barrier regarding the HPV vaccine, which could affect the HPV vaccine mandate. Consistent with our findings, two studies that evaluated a group of Latina mothers found a low level of knowledge about cervical cancer, HPV infection, and HPV vaccine [21, 22]. Gualano et al. [25] highlighted that limited knowledge on HPV vaccine could be associated with the opposition of the mandatory HPV vaccination. Despite this limitation, our participants expressed their interest in knowing about the pros and cons of the requirement of HPV vaccine for school entrance, duration of vaccine effectiveness, and potential side effects to make an informed decision about vaccinating against HPV. Studies have shown this same parents’ interest for more information before considering vaccinate their child [20–22, 39].

Some of our participants expressed limited access to the HPV vaccine. A possible explanation of this limitation was the long-term recovery of several immunization clinics after the impact of Hurricane Irma and Hurricane María in PR [40]. Barriers regarding immunization exemptions are poorly studied topics. The participants who required the exemption in our study mentioned the difficulties of the process and the cost. The DOH has a strict exemption requirement as it only allows medical and religious exemption for vaccination (no philosophical exemption) [14]. The rate of exemption in PR is still unknown. Further efforts need to explore if exemption requests have increased in PR due to the implementation of the HPV vaccine since August 2018 and how (if any) impacted the uptake of other adolescent vaccines over time.

### Strengths and limitations

Mandatory vaccination for school entry is a strategy to increase vaccination uptake, make catch-up, or reduce the gap between vaccine groups, including the HPV vaccine [41]. To our knowledge, this is the first qualitative study in PR that describes parental perspectives about the HPV vaccine school entry requirement and portrays challenges in the exemption process. Our study is one of two studies [10] that evaluated the parental perspective about the HPV vaccine policy enacted on a US territory. Regardless of this effort, our research has several limitations worth discussing. The size of the sample and convenience sampling did not allow for the generalization of the results. In addition, a population-level profile of those parents who delay or reluctant to refuse the HPV vaccination in PR does not exist. Our sample is similar in education level, income, and health insurance compared to the Puerto Rican general population [42]. Despite several outreach and community engagement efforts performed, we recruited a relatively small sample of participants. The narrow eligibility requirements made this a hard-to-reach population for recruitment, given that PR has a high HPV vaccine initiation rate (75.7%) among teenagers [43]. Participants’ time availability and concerns of being exposed to the public spotlight (due to their views on vaccination) were barriers to recruitment. However, we highlighted those in-person recruitment strategies (e.g., visiting public housing complexes and distribute flyers in activities, both with the opportunity to do a brief discussion) were more effective (17/20 participants) than Facebook or flyers from a desk. For some participants, in-depth interview was more comfortable because they could open up more about their view of the school policy and HPV vaccine. We opened our recruitment for female and male parents; however, only females participated. Understand the fathers’ perception could help us getting additional insight on vaccines and this new requirement and understand potential divergent family dynamics in the decision of vaccinating (or not) their children against HPV [44].

## Conclusion

We conducted this study in a timely opportunity to understand the parental perceptions regarding the implementation of this recent HPV vaccine requirement. Most of the barriers identified could be modifiable addressing with targeted educational programs with parents, school staff and providers, health communication strategies across agencies responsible for the implementation, public acceptability, and political acceptability to support this policy [29, 31, 45]. According to unpublished preliminary results using data of 2010 through 2019 provided by the Puerto Rico Immunization Registry and our research team, an increase of 54% was observed in HPV vaccination initiation rate (not completion) for 11–12 years old children after implementing the requirement [46]. However, it is unknown how the COVID-19 pandemic has impacted the parental perception of the school required vaccines and HPV vaccine uptake. Future research could explore target educational interventions that might help address and boost parental concerns, maintain a high rate of the first dose, and increase completion and adherence to the HPV vaccine schedule of the second dose. Also, it is important to understand the barriers between a parent who refuse in comparison to a parent who delayed the vaccination could be differents [47]. Understanding the progression of parental perceptions throughout the policy implementation, and how current scenarios might impact their decisional process should help us to develop effective interventions that respond to the identified barriers and facilitators in the future.

## Supplementary information

**Additional file 1.** GUÍA DEL MODERADOR: ENTREVISTA A PADRES O ENCARGADOS. Description of data: This document is the guideline questions used for the focus group and in-depth interview in our study.

## Data Availability

The data sets generated and analyzed during the current study are not publicly available due to our confidentiality commitment to our participants to keep such data in our offices, but they are available from the corresponding author on reasonable request and after being discussed with the IRB.

## Abbreviations

HPV: human papillomavirus
WHO: World Health Organization
US: United States of America
ACIP: Advisory Committee on Immunization Practices
PR: Puerto Rico
SHPR: Puerto Rico Secretary of Health.
DOH: Puerto Rico Department of Health
