# Supplementary material for: Understanding parents’ views toward the newly enacted HPV vaccine school entry policy in Puerto Rico: a qualitative study
Source: BMC Public Health. 2021 Oct 25;21:1938. doi: 10.1186/s12889-021-11952-w (PMC8544631; doi:10.1186/s12889-021-11952-w)
Supplement: Supplementary file 1 — Additional file 1. GUÍA DEL MODERADOR: ENTREVISTA A PADRES O ENCARGADOS. Description of data: This document is the guideline questions used for the focus group and in-depth interview in our study. [file 12889_2021_11952_MOESM1_ESM.pdf]

COMPREHENSIVE CANCER CENTER OF THE UNIVERSITY OF PUERTO RICO &
UNIVERSITY OF PUERTO, BIOMEDICAL SCIENCES CAMPUS

MODERATOR GUIDE: PARENT OR GUARDIAN INTERVIEW

MODULE B

MODERATOR: Read the introduction to the participants:

- *Hello, my name is ________________________ and I work for the Comprehensive Cancer Center of the University of Puerto Rico. With me today is __________________________, who also works for the Comprehensive Cancer Center and will be taking notes during this activity.*
- *We are here to gather your opinions regarding the human papilloma virus vaccine (also known as HPV), and the most recent decisions about the public policy on the island.*
- *We are interested in obtaining information about the knowledge of HPV and the vaccination policy that influences the practice or resistance of vaccination against HPV among adolescents 11-12 years old in Puerto Rico.*

MODERATOR: Read the consent form to the participants and ask them to please sign. Collect the signed forms and read the following basic rules:

Now I would like to review with you some of the basic rules for today's group discussion:

- *We will be hearing very diverse opinions, please respect others opinions even if you do not agree. Please know it is okay to express your opinion, despite it being different from what other participants have said.*
- *Please speak one at a time, so we all have an opportunity to speak and be heard clearly in the recording.*
- *If you all speak loud and clear, you will be heard without difficulty and I will not need to repeat what was said for the recording.*

MODERATOR: Make sure there are no doubts regarding the basic rules prior to continuing with the icebreaker.

ICEBREAKER

Since most of us don't' know each other, we will begin by introducing ourselves. We will go around the room, state your first name and name an activity or hobby you like to do for fun in your spare time.

MODERATOR: After they have all introduce themselves, continue by reading the following…

Thank you for introducing yourselves. Now we will begin recording this session.

TURN ON VOICE RECORDERS

MODERATOR: Read the following introduction and ask the following questions.

I will begin by asking several questions regarding the public vaccination policy in Puerto Rico.

I. VACCINATION POLICY AGAINT HPV IN PUERTO RICO

KNOWLEDGE AND ATTITUDES REGARDING VACCINATION

1. **What is your opinion about vaccination (vaccines) in general?**

2. **What do you know about the human papilloma virus vaccine?**
   Elaborate: What have you heard about this vaccine in each of these mediums? (Stories from family or friends, school, social media, television, newspaper, radio, health care professionals)
   Elaborate: Benefits or risks?

In August of 2018, Puerto Rico implemented a public policy to include the Human Papilloma Virus vaccine or HPV, as part of the requirement for school entry, in children between the ages of 11-12 years old.

KNOWLEDGE AND ATTITUDES REGARDING THE PUBLIC POLICY

3. **How did you hear about this policy?**
   Elaborate: (school, clinic, physician, family members, friends, social media, television, newspaper, radio)
   Elaborate: What have you heard about this policy in each of these sources?

4. **What is your opinion regarding this public policy?**
   Elaborate: Do you agree or disagree with this policy? Why?
   Elaborate: How prepared did you felt at the time you learned this information to make a decision regarding vaccinating your child?

5. **How necessary do you believe the HPV vaccine is as a mandatory vaccination in schools?**

6. **What do you think about the exemption policy for vaccines?**
   Elaborate: Is it beneficial or a drawback?
   Elaborate: Are you aware of the exemptions that apply in Puerto Rico (religious and medical)?
   Elaborate: Are you aware of the process and forms to request exemptions?
   Elaborate: How did you learn about exemptions and the processes they entail?

PRACTICES AND PROCEDURES

7. **Prior to the beginning of the school year, did you received information regarding this policy (the need to vaccinate your child against HPV as part of the new vaccination requirements) at the school your child attends?**
   Elaborate: How did the school communicate the requirements for the new school year? (letter, email, public bulletin)?
   Elaborate: What information did you receive regarding the new requirement?
   Elaborate: Did you understand the information provided?
   Elaborate: What questions or concerns did you have the moment you received this information?

8. **Why have you decided not to vaccinate your child against HPV before the beginning of the school year in August of 2018?**
   Elaborate: What were the reasons for not vaccinating your child prior to the school year? (Lack of information, lack of physician advice, young age for the vaccine, religious reasons, effectiveness of the vaccine, experimentation, side effects, pharmaceutical agenda).
   Elaborate: family lifestyles, risk perceptions to the immune system, risk of illness, vaccine efficacy, side effects, negative experience with vaccination, social media, influential community members.

II. INFORMATION ABOUT HPV AND THE VACCINE

INTERNAL INFLUENCES

9. **What information do you consider important and are interested to be included in messages to promote the vaccination policy for children between the ages of 11 and 12 years old?**
   Elaborate: Knowing the time limit to vaccinate my child, knowing the cost of the vaccine, knowing if my insurance plan covers the vaccine, knowing where I can vaccinate my child, knowing the side effects, knowing how many doses?
   Elaborate: Who would you like to hear these messages? (Puerto Rico Secretary of Health, parents who experienced vaccination, other).
   Elaborate: What is the best way to make the information known? (e.g., Schools, radio, TV ad, through social media platforms)

WILLINGNESS TO CHANGE

10. **As parents, what messages about this policy (order, regulation) would further motivate you to make the decision to vaccinate your child against HPV in time, prior to beginning sixth grade?**
    Elaborate: Information sources (telephone, webpage, flyers)
    Elaborate: In your opinion, whom should guide parents such as yourselves, regarding the policy (the Puerto Rico Department of Education, Puerto Rico Department of Health)?

WRAP UP

Is there anything else we did not discuss you would like to share or that we should know?

CONCLUSION

I want to thank you all for taking the time and sharing with us today. We have enjoyed this meeting and you have provided a lot of information.
